# A New Reconstructive Technique of the Anterolateral Ligament with Iliotibial Band-Strip

**DOI:** 10.2174/1874325001711010321

**Published:** 2017-04-27

**Authors:** Bart Stuyts, Elke Van den Eeden, Jan Victor

**Affiliations:** 1 Sint Augustinus Hospital, Oosterveldlaan 24, 2610 Wilrijk, Belgium; 2 University Hospital Gent, Ghent, Belgium

**Keywords:** Anterior cruciate ligament, Anterolateral ligament, Iliotibial band, Internal rotation, Extra-articular tenodesis, Intra-articular reconstruction

## Abstract

**Background:**

Anterior cruciate ligament (ACL) reconstruction is a well-established surgical procedure for the correction of ACL ruptures. However, the incidence of instability following ACL reconstruction is substantial. Recent studies have led to greater insight into the anatomy and the radiographic characteristics of the native anterolateral ligament (ALL), along with its possible role in residual instability after ACL reconstruction.

**Method:**

The current paper describes a lateral extra-articular tenodesis to reconstruct the ALL during ACL procedures, using a short iliotibial band strip. The distal insertion of this strip is left intact on the anterolateral side of the proximal tibia, and the proximal part is fixed at the anatomic femoral insertion of the ALL.

**Results:**

Our technique avoids the sacrifice of one of the hamstring tendons for the ALL reconstruction. Additionally, there is no interference with the anatomical location or function of the LCL.

**Conclusion:**

Our technique offers a minimally invasive and nearly complete anatomical reconstruction of the ALL with minimal additional operative time.

## INTRODUCTION

Anterior cruciate ligament (ACL) reconstruction is a well-established surgical procedure for the correction of ACL ruptures [1]. However, the incidence of grade II pivot shift following ACL reconstruction is almost 20% following ACL reconstruction with either hamstring or patellar tendon graft [2]. The state of a key anterolateral stabilizing structure of the knee, the anterolateral ligament (ALL), may be responsible for the unstable, unsatisfactory results following ACL reconstruction [3,4]. The ALL, which is located in the anterolateral portion of the knee and adjacent to the joint capsule, has been reported to be present in 50% [5] to 96% [6] of all knee joints studied, yet the functional importance of ALL in activities of daily living and exercise still requires elucidation [3,4, 7, 8].

Extra-articular ligament reconstruction techniques that address the ALL may potentially confer rotational stability and restore function to the knee [3,7]. Patients who present anterolateral and rotator knee instability, or patients who have recalcitrant instability despite standard ligament reconstruction would particularly benefit from such a combined approach [4].

In the decades before the studies on the ALL's anatomy and its biomechanical function were published, there was literature describing several extra-articular techniques to reconstruct the ALL [9, 10]. A meta-analysis of outcome studies describing combined lateral extra-articular tenodesis (LEAT) and intra-articular ACL reconstruction confirmed that LEAT is effective in terms of reduction of pivot shift [11]. However, many techniques reconstruct the ALL in a non-anatomic manner or interfere with the anatomy and function of the lateral collateral ligament (LCL).

In this article, we present a technique with a short autologous iliotibial band (ITB)-strip fixed proximally at the anatomic femoral origin of the ALL, which does not interfere with the anatomy and function of the LCL.

Ethics committee approval was obtained. The patient was informed that photographs from the surgical procedure would be submitted for publication, and his consent was received.

## TECHNIQUE

**Fig. (1):**
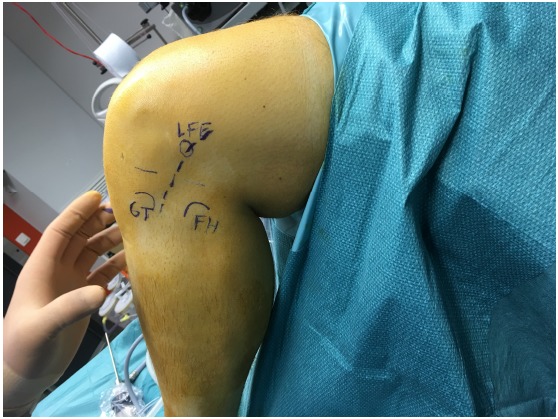
Anatomic landmarks for the anterolateral ligament are indicated. Abbreviations: LFE, lateral femoral epicondyle; GT, Gerdy tubercle; FH, fibular head.

**Fig. (2):**
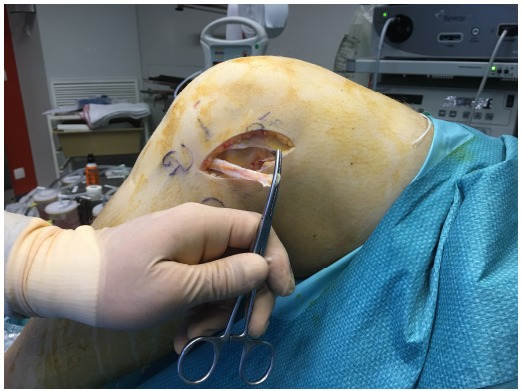
Harvesting of the iliotibial band strip is shown.

**Fig. (3):**
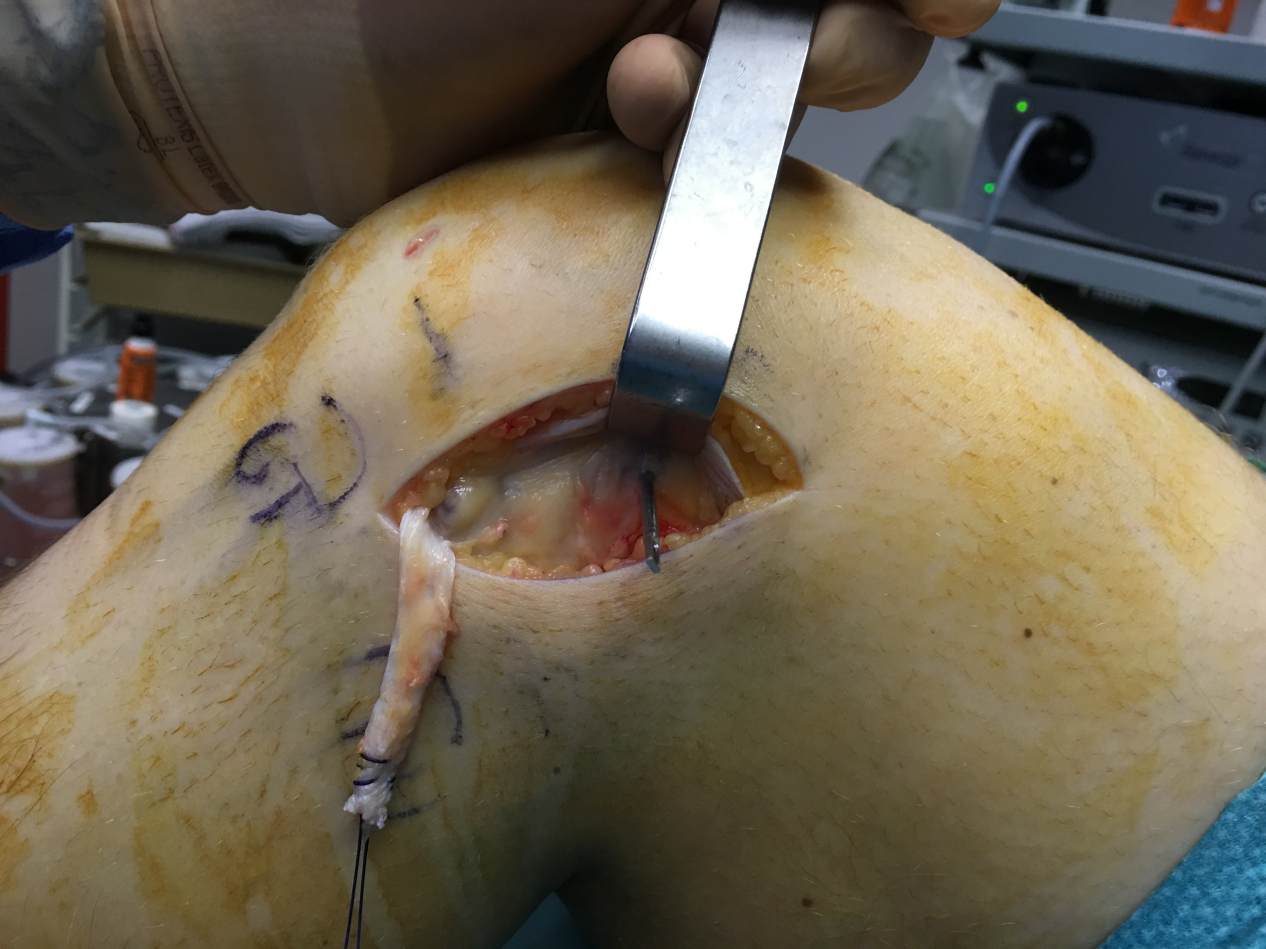
Drilling with the use of a guide-wire at the femoral origin of the anterolateral ligament is shown.

**Fig. (4):**
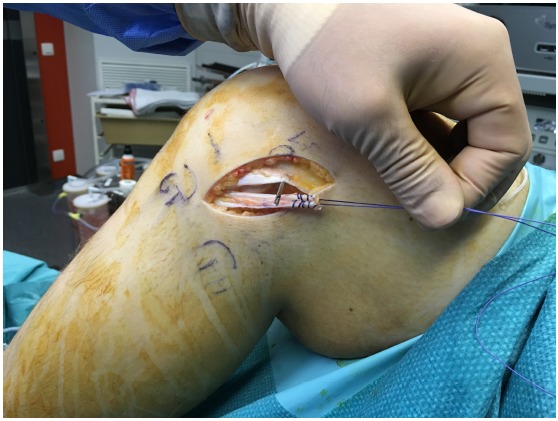
The proximal end of the iliotibial band strip is prepared.

**Fig. (5):**
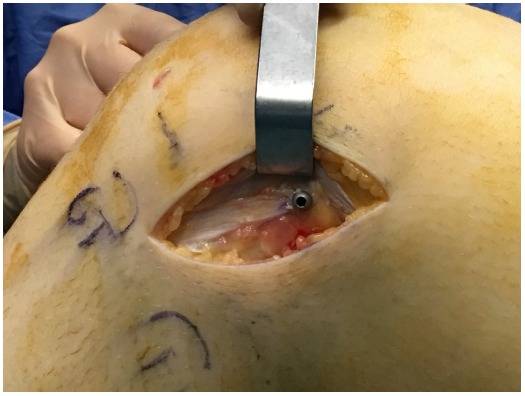
Fixation of the iliotibial band strip in the femoral tunnel is shown.

The patient is placed in a supine position on the operating table. A tourniquet is placed high on the thigh and inflated. ACL reconstruction is performed in a conventional arthroscopic manner with the use of autologous hamstring graft. For the additional LEAT, the anatomic landmarks are drawn: Gerdy’s tubercle (GT), the fibular head (FH), and the lateral femoral epicondyle (LFE) (Fig. **1**). A skin incision of about 7 cm is made starting just posterior to the GT to 1.5 cm proximal to the LFE. An 8 mm ITB strip is taken from the posterior third of the ITB (Fig. **2**). The distal insertion of the strip is left intact on the anterolateral tibia. Proximally, the strip is cut 2 cm proximal to the LFE. The anatomical femoral insertion of the ALL on the prominence of the lateral femoral epicondyle, slightly anterior to the socket from which the LCL originated [3] and proximal and posterior to the insertion of the popliteus tendon, is identified by palpation or with the aid of fluoroscopy if needed. A guide wire is drilled at the femoral origin of the ALL from the lateral side to the medial side (Fig. **3**). The proximal end of the ITB-strip is cut 1.5 cm proximal to this point and then whipstitched with a Vicryl® 1 suture wire (Ethicon, Somerville, NJ) (Fig. **4**). A drill hole with a depth of 20 mm and a diameter of 6 mm is made over this guide wire. The sutures are passed with the guide wire to the medial side. They are tensioned at the medial side at a 60° to 90° angle of knee flexion, and a 10° to 15° angle of external rotation of the foot in accordance with studies that have shown higher tensioning of this structure with increased knee flexion [12,13]. The ITB-strip is then fixed in the femoral tunnel with a metal interference screw with a diameter of 6 mm (Fig. **5**). The ITB window, subcutaneous tissues, and skin are closed.

## DISCUSSION

The knee, as the largest and most complex joint in the body, is prone to injury. Yet, an understanding of the function of its anatomic structures has not been fully established. The ALL was once considered a thickening of the joint capsule or fibrous tissue around the knee [4]. Segond described the ‘pearly, resistant, fibrous band’ at the anterolateral aspect of the human knee, attached to the eponymous Segond fracture [3, 14]. Segond fractures are predictive for ACL ruptures [4, 7]. However, traditionally, reconstruction of the unstable knee has focused on the restoration of the cruciate and collateral ligaments [4]. More recently, the ALL has been defined as a distinct anatomic structure that confers rotational stability to the knee. Consistent radiographic landmarks have been established for the origin and insertion of ALL, which are of importance in the surgical reconstruction [15-17]. The physiologic importance of ALL has led to the refinement of surgical techniques in the anatomic arthroscopic reconstruction of the ACL, thus imparting the restoration of kinematics of the native knee and its ligaments [16].

The physical properties of the ALL in relation to the ACL and LCL confer its role in rotation of the knee. The anterior insertion of the ALL in relation to the LCL provides anterior and rotational stability [8]— particularly at higher angles of flexion [13]. A mechanically favorable lever arm is created by the lateral position of the ALL with respect to the ACL, which resists rotator movement [4]. However, the tensile strength of the ALL is only modest when compared to the cruciate ligaments [16, 18]. In cadaver specimens, these properties have been clearly demonstrated *via* comparison of rotational laxity before and after resection of the ALL and by measuring grafts with modified attachment points during knee flexion [19].

For revision ACL reconstruction, in patients with high-grade rotational instability, and in patients competing in high-pivoting sports, a combined ALL and ACL reconstruction is a recommended surgical option [4].

We present a new technique for an ALL reconstruction using a short ITB-strip. The distal insertion of this strip is left intact on the anterolateral side of the proximal tibia and the proximal part is fixed at the anatomic femoral insertion of the ALL. By 1967, Lemaire had already described a technique that used a comparably thick ITB strip of 1.5 cm times 16 cm that also preserved the anatomic insertion of the ITB on the tibia [20]. In contrast to our technique, on the femoral side, Lemaire inserted the graft under the origin of the LCL. The authors of the current technique believe that this may cause interference with the anatomy and function of LCL.

More recently, Smith *et al.* used a gracilis tendon autograft to reconstruct the ALL in conjunction with “all-inside” quadrupled semitendinosus ACL reconstruction [4].

The advantages of our technique are that an autologous graft is used without sacrificing one of the hamstring tendons for the ACL reconstruction. This technique does not interfere with the anatomical location or function of the LCL. It offers a minimally invasive and nearly anatomical reconstruction of the ALL with minimal additional operative time and morbidity.

We believe ACL and ALL reconstruction must be considered in patients presenting with ACL rupture with high-grade rotational instability, such as in elite athletes or those with hypermobility, and in revision ACL reconstruction. A clinical study is planned to demonstrate the efficacy and safety of this new technique in patients.

## References

[1] Desai N., Bjornsson H., Samuelsson K., Karlsson J., Forssblad M. (2014). Outcomes after ACL reconstruction with focus on older patients: Results from The Swedish National Anterior Cruciate Ligament Register.. Knee Surg. Sports Traumatol. Arthrosc..

[2] Mohtadi N.G., Chan D.S., Dainty K.N., Whelan D.B. (2011). Patellar tendon versus hamstring tendon autograft for anterior cruciate ligament rupture in adults.. Cochrane Database Syst. Rev..

[3] Claes S., Vereecke E., Maes M., Victor J., Verdonk P., Bellemans J. (2013). Anatomy of the anterolateral ligament of the knee.. J. Anat..

[4] Smith J.O., Yasen S.K., Lord B., Wilson A.J. (2015). Combined anterolateral ligament and anatomic anterior cruciate ligament reconstruction of the knee.. Knee Surg. Sports Traumatol. Arthrosc..

[5] Stijak L., Bumbasirevic M., Radonjic V., Kadija M., Puskas L., Milovanovic D., Filipovic B. (2016). Anatomic description of the anterolateral ligament of the knee.. Knee Surg. Sports Traumatol. Arthrosc..

[6] Van der Watt L., Khan M., Rothrauff B.B., Ayeni O.R., Musahl V., Getgood A., Peterson D. (2015). The structure and function of the anterolateral ligament of the knee: A systematic review.. Arthroscopy.

[7] Claes S., Luyckx T., Vereecke E., Bellemans J. (2014). The Segond fracture: A bony injury of the anterolateral ligament of the knee.. Arthroscopy.

[8] Dodds AL, Halewood C, Gupte CM, Williams A, Amis AA (2014). The anterolateral ligament: Anatomy, length changes and association with the Segond fracture.. Bone Joint J..

[9] Dodds A.L., Gupte C.M., Neyret P., Williams A.M., Amis A.A. (2011). Extra-articular techniques in anterior cruciate ligament reconstruction: A literature review.. J. Bone Joint Surg. Br..

[10] Roessler P.P., Schuttler K.F., Heyse T.J., Wirtz D.C., Efe T. (2016). The anterolateral ligament (ALL) and its role in rotational extra-articular stability of the knee joint: A review of anatomy and surgical concepts.. Arch. Orthop. Trauma Surg..

[11] Hewison C.E., Tran M.N., Kaniki N., Remtulla A., Bryant D., Getgood A.M. (2015). Lateral Extra-articular Tenodesis Reduces Rotational Laxity When Combined With Anterior Cruciate Ligament Reconstruction: A Systematic Review of the Literature.. Arthroscopy.

[12] Helito C.P., Helito P.V., Bonadio M.B., da Mota E.A., Bordalo-Rodrigues M., Pecora J.R., Camanho G.L., Demange M.K. (2014). Evaluation of the length and isometric pattern of the anterolateral ligament with serial computer tomography.. Orthop. J. Sports Med..

[13] Parsons E.M., Gee A.O., Spiekerman C., Cavanagh P.R. (2015). The biomechanical function of the anterolateral ligament of the knee.. Am. J. Sports Med..

[14] Segond P. (1879). Recherces et expérmintales sur les épanchements sanguins du genou par entorse.. Progr. Med. (Paris).

[15] Helito C.P., Demange M.K., Bonadio M.B., Tirico L.E., Gobbi R.G., Pecora J.R., Camanho G.L. (2014). Radiographic landmarks for locating the femoral origin and tibial insertion of the knee anterolateral ligament.. Am. J. Sports Med..

[16] Kennedy M.I., Claes S., Fuso F.A., Williams B.T., Goldsmith M.T., Turnbull T.L., Wijdicks C.A., LaPrade R.F. (2015). The anterolateral ligament: An anatomic, radiographic, and biomechanical analysis.. Am. J. Sports Med..

[17] Rezansoff A.J., Caterine S., Spencer L., Tran M.N., Litchfield R.B., Getgood A.M. (2015). Radiographic landmarks for surgical reconstruction of the anterolateral ligament of the knee.. Knee Surg. Sports Traumatol. Arthrosc..

[18] Zens M., Feucht M.J., Ruhhammer J., Bernstein A., Mayr H.O., Südkamp N.P., Woias P., Niemeyer P. (2015). Mechanical tensile properties of the anterolateral ligament.. J. Exp. Orthop..

[19] Kittl C., Halewood C., Stephen J.M., Gupte C.M., Weiler A., Williams A., Amis A.A. (2015). Length change patterns in the lateral extra-articular structures of the knee and related reconstructions.. Am. J. Sports Med..

[20] Lemaire M. (1967). Rupture ancienne du ligament croisé antérieur du genou.. J. Chir. (Paris).

